# Vaccination with Combination DNA and Virus-Like Particles Enhances Humoral and Cellular Immune Responses upon Boost with Recombinant Modified Vaccinia Virus Ankara Expressing Human Immunodeficiency Virus Envelope Proteins

**DOI:** 10.3390/vaccines5040052

**Published:** 2017-12-19

**Authors:** Sailaja Gangadhara, Young-Man Kwon, Subbiah Jeeva, Fu-Shi Quan, Baozhong Wang, Bernard Moss, Richard W. Compans, Rama Rao Amara, M. Abdul Jabbar, Sang-Moo Kang

**Affiliations:** 1 Department of Microbiology and Immunology, Emory Vaccine Center, Emory University School of Medicine, 1510 Clifton Rd., Atlanta, GA 30322, USA; sgangad@emory.edu (S.G.); rcompan@emory.edu (R.W.C.); ramara@emory.edu (R.R.A.); ajabbar@emory.edu (M.A.J.); 2 Center for Inflammation, Immunity & Infection, Institute for Biomedical Sciences, Georgia State University, Atlanta, GA 30303, USA; ymankwon@gmail.com (Y.-M.K.); jsubbiah@gsu.edu (S.J.); bwang23@gsu.edu (B.W.); 3 Department of Medical Zoology, Kyung Hee University School of Medicine, Seoul 130-705, Korea; fquan01@gmail.com; 4 Laboratory of Viral Diseases, National Institute of Allergy and Infectious Diseases, National Institutes of Health, Bethesda, MD 20892, USA; BMOSS@niaid.nih.gov

**Keywords:** HIV vaccines, DNA, VLPs, recombinant MVA

## Abstract

Heterologous prime boost with DNA and recombinant modified vaccinia virus Ankara (rMVA) vaccines is considered as a promising vaccination approach against human immunodeficiency virus (HIV-1). To further enhance the efficacy of DNA-rMVA vaccination, we investigated humoral and cellular immune responses in mice after three sequential immunizations with DNA, a combination of DNA and virus-like particles (VLP), and rMVA expressing HIV-1 89.6 gp120 envelope proteins (Env). DNA prime and boost with a combination of VLP and DNA vaccines followed by an rMVA boost induced over a 100-fold increase in Env-specific IgG antibody titers compared to three sequential immunizations with DNA and rMVA. Cellular immune responses were induced by VLP-DNA and rMVA vaccinations at high levels in CD8 T cells, CD4 T cells, and peripheral blood mononuclear cells secreting interferon (IFN)-γ, and spleen cells producing interleukin (IL)-2, 4, 5 cytokines. This study suggests that a DNA and VLP combination vaccine with MVA is a promising strategy in enhancing the efficacy of DNA-rMVA vaccination against HIV-1.

## 1. Introduction

Over 40 million lives have been claimed due to the human immunodeficiency virus (HIV) pandemic around the world, and the numbers of HIV infection keep increasing. Vaccination can be the most cost-effective measure for the control of HIV transmission, particularly in the developing countries. Early clinical trials using Vaxgen recombinant gp120 HIV Env protein failed to induce protective immune responses [1]. Soluble HIV Env protein vaccines were not effective in inducing cytotoxic CD8 T cell immunity or protective levels of neutralizing antibodies. Because of the safety concerns, the approaches for using inactivated virus or live attenuated HIV vaccines would not be appropriate for application to humans [2]. The failure of the Merck HIV Vaccine Trials Network 502 Phase IIb STEP trial required new efforts and approaches in developing effective HIV vaccines [3,4,5]. A clinical trial of Thai AIDS vaccines indicated the first positive results by vaccination composed of two prime injections with a recombinant live canarypox vector and two booster injections with soluble gp120 subunit vaccines [6]. However, the results from the Thai AIDS vaccine clinical trial showed a marginal benefit, indicating that development of improved vaccines should be given a high priority.

Antibody-mediated protection against viral infections involves multiple mechanisms. Neutralizing antibodies can block the entry of virus into target cells. Coating of virions with antibodies facilitates virus uptake and destruction by phagocytes. Infected cells containing bound antibodies can be eliminated by natural killer cells by antibody-dependent cellular cytotoxicity [7,8]. Binding of complement to antibodies on virions or infected cells can also initiate complement-mediated lysis or opsonization [9,10,11].

DNA and recombinant modified vaccinia virus Ankara (rMVA) vaccines are a candidate HIV vaccine inducing both humoral and cellular immunity [12,13]. A phase 1 test of DNA and rMVA HIV vaccines demonstrated safety and immunogenicity of inducing B cells and T cells in most of vaccinees [14], which is now further in clinical trials. Cytotoxic CD8 T cells are known to control HIV infection by killing infected cells, suppressing viral replication and entry by secreting antiviral cytokines and chemokines [15,16,17]. Helper CD4 T cells are required for B cells to undergo germinal center reactions leading to IgG hypermutation and affinity maturation.

Developing a vaccine capable of inducing both protective humoral and cellular immune responses is a central challenge for the development of an effective HIV vaccine. A DNA plasmid vaccine expressing granulocyte-macrophage colony-stimulating factor (GM-CSF) has been shown to enhance protective humoral responses by DNA-rMVA prime boost vaccination [18,19,20]. DNA and rMVA vaccines induce immune responses via direct in vivo synthesis of antigens. In contrast, virus-like particles (VLPs) can directly present native structures of HIV Env proteins to the immune system and stimulate dendritic cells (DCs) as professional antigen presenting cells required for CD4 and CD8 T cell activation. Therefore, we hypothesized that HIV VLPs would enhance immunogenicity of DNA-rMVA vaccines. Here we investigated humoral and cellular immune responses in mice after sequential immunizations with DNA, a combination of DNA + VLP, and rMVA. This study revealed that use of a combination of DNA and VLP vaccine as the first boost in the context of DNA-rMVA prime boost immunization could provide significantly enhanced humoral IgG antibody responses together with T cell immune responses.

## 2. Materials and Methods

### 2.1. Preparation of DNA, rMVA Expressing tPAhFLex-gp120, and VLP Vaccines

**Figure 1:**
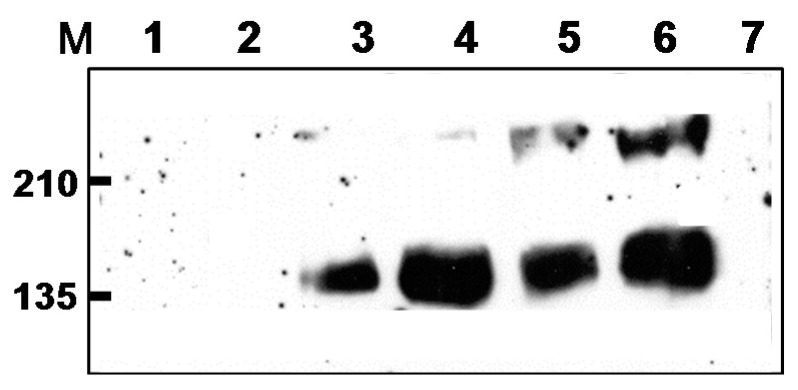
Expression of hFlex-gp102 in rMVA-infected cell lysates. Western blot analysis of DF-1cell lysates infected with rMVA expressing tPAhFlex-gp120 after probing with anti-hFlex antibody. M: molecular weight standard marker, 1–2: Negative orientation of rMVA constructs; 3–4: Positive orientation of rMVA tPAhFlex-gp120; 5–6: positive controls (hFlex-gp120 proteins); 7: MVA vector control.

The DNA vaccine is a plasmid pNGVL expression vector with tPAhFLex-gp120 which expresses HIV-1 (89.6) gp120 proteins with the extracellular domain of Flt3 ligand as previously described [21]. A gene fragment (2Kb) encoding HIV-1 (89.6) gp120 conjugate of the tPA (tissue plasminogen activator) signal and Flt3 ligand (tPAhFLex-gp120) [21] was cloned into the transfer vector pTH5loxGZ to generate rMVA as previously described [22,23,24]. DF-1 cells (chicken embryo fibroblasts) were transfected with plasmid pTH5loxGZ containing tPAhFLex-gp120 using the EFFECTINE kit (Qiagen, Germantown, MD, USA) according to manufacturer’s instructions, and DF-1 cell lysates were screened for the expression of hFlex-gp120 by western blot (Figure 1) using anti-hFlex antibody (R&D systems, Minneapolis, MN, USA) [21]. HIV VLP vaccines presenting HIV-1 89.6 Env proteins were produced in Spodoptera frugiperda Sf9 insect cells as described [25,26]. Briefly, insect cells were co-infected with recombinant baculoviruses (rBV) expressing HIV-1 89.6 and Gag proteins. After 3 days, the culture supernatants containing released HIV VLPs were harvested by low speed centrifugation and then subjected to ultracentrifugation to precipitate VLP vaccines. Sucrose step-gradient ultracentrifugation was applied to further purify HIV VLPs as described in [25,26].

### 2.2. Mice and Immunizations 

Female BALB/c mice (6–8 weeks old) were obtained from Charles River and maintained in the vivarium at the Yerkes National Primate Research Center according to the institutional animal care (IACUC) guidance. Age-matched mice were used for DNA-VLP-MVA vaccination experiments. Groups of mice (*n* = 10) were subcutaneously prime immunized with 200 µL PBS buffer containing 50 µg of plasmid DNA vaccine (pNGVL tPAhFLex-gp120), and then boosted (1st) with DNA (50 µg), HIV VLP (50 µg), or a combination of HIV VLP (50 µg) and DNA (50 µg) (VD). The second boost immunization was followed by DNA vaccine for the D-D-D group or rMVA (1 × 10^7^ plaque forming units, PFU) for the D-D-M, D-V-M, and D-VD-M groups. Each three immunizations were carried out with a 4-week interval and bloods drawn after 12 days of each vaccination. 

### 2.3. Preparation of Peripheral Blood Mononuclear Cells (PBMCs) and Intracellular Cytokine Staining (ICC) Assay 

Blood samples were drawn into the tubes containing heparin (5 U/mL) in PBS. Histopaque (1 mL at 37 °C) were under-layered and spun at 2000 rpm (20 °C) for 20 min. The interphase containing PBMCs was collected, washed, and suspended in complete media (DMEM containing 10% fetal calf sera). For ICC assays, PBMCs (10^5^–10^6^ cells/well) were stimulated in vitro in the presence of gp120 V3 loop peptide (0.1 µg/mL, IGPGRAFYAR), restricted by H2-D^d^ in BALB/c mice [27,28] or a pool of Env peptides (15mers with 11aa overlap, NIH AIDS reagent program) with brefeldin A (Golgiplug: BD Biosciences-US, San Jose, CA, USA). After stimulation for 6 h, the cells were stained with FITC-conjugated anti-CD3 (clone: 17A2) (BD Biosciences-US, San Jose, CA, USA) and PerCP labeled anti-CD8 (clone: 53–6.7) antibodies. After washing, the cells were permeabilized and stained with APC-labeled rat anti-mouse IFN-γ (clone: XMG1.2; BD Biosciences-US) for intracellular IFN-γ with the Cytofix/Cytoperm staining kit (BD Biosciences-US) according to the manufacturer’s recommendations. 

### 2.4. DC Preparation and CD4^+^ T Cell Isolation

In vivo expansion of DCs in BALB/c mice was carried out by injecting plasmid DNA that expresses human Flt3 ligand extracellular domain (hFLex) as described [21]. After nine days of hFlex DNA intravenous injection, spleens were separated. Single cell suspensions after treating with Type 4 collagenase (Worthington Biochemical, Lakewood, NJ, USA) were incubated with CD11c (N418) microbeads and DCs purified by passing through the columns according to manufactures instructions (Miltenyi Biotec Inc., San Diego, CA, USA). Purified DCs were used to stimulate CD4^+^ T cells from immunized mice in the presence of peptides. CD4^+^ T cells were isolated from single cell suspensions of spleens from the different groups of mice using a CD4^+^ isolation kit (Miltenyi Biotec Inc.) according to manufacturer’s instructions. CD4^+^ T cells were stimulated by either single peptide or pool of peptides in the presence of DCs at a DC:CD4 T cell ratio of 1:10 for proliferation, cytokine production and ELISPOT assays.

### 2.5. Lymphoproliferation and Cytokine Production In Vitro 

The spleen cells (1 × 10^6^) were stimulated in vitro for lymphoproliferation with either a single peptide (0.2 µg/mL) as described above or a pool of 89.6 Env peptides (1 µg/mL, 15mers with 11aa overlap, NIH AIDS reagent program). Stimulation was carried out for 72 h, and tritiated [H] thymidine used for 12 h before harvesting the cells. Stimulation for cytokine assays was carried out for 12–36 h by using 1 million cells/mL with either single or a pool of Env peptides. Isolated CD4^+^ cells (1 × 10^5^) were stimulated in the presence of DCs (1 × 10^4^) for lymphoproliferation or cytokine production. The stimulated cell culture supernatants were assayed for cytokines IL-2, IL-4, IL-5, and IFN-γ using cytokine assay kits from either BD Biosciences-US (San Jose, CA, USA) or eBiosciences (Thermo Fisher Scientific, Norcross, GA, USA) according to manufacturer’s instructions.

### 2.6. ELISA (Enzyme-Linked Immunosorbent Assay)

To determine anti-gp120 antibody titers, we coated the maxisorb plates with 89.6 gp120 Env-His purified in house using recombinant vaccinia virus and His-Tag column as described [21]. Serially diluted sera were added to the HIV-1 gp120 antigen coated plates for incubation, followed by horse radish peroxidase (HRP) conjugated anti-mouse IgG and then TMB substrate (PIERCE) to develop color (450 nm). An ELISA titer was defined as the reciprocal of the serum dilution showing an optical reading three times higher than the naive sera. 

### 2.7. ELISPOT (Enzyme-Linked ImmunoSpot)

MultiScreen 96 well sterile filter plates (Millipore, Burlington, MA, USA) were coated overnight with rat anti-mouse IFN-γ antibody (clone R4-6A2, BD Biosciences-US), rat anti-mouse IL-2 antibody (clone JES6-1A12, BD Biosciences-US), or rat anti-mouse IL-4 antibody (clone BVD4-1D11, BD Biosciences-US) in 100 µL (4 µg/mL) bicarbonate buffer (pH 9.6). Whole splenocytes (1 × 10^6^) or CD4^+^ T cells (1 × 10^5^) plus DCs (1 × 10^4^) were added into each well in the presence or absence of MHC class I peptide (0.2 µg/mL) or a pool of peptides (1 µg/mL). After 36 h culture, the plates were washed and incubated overnight with biotinylated rat anti mouse IFN-γ (clone XMG 1.2), biotinylated rat anti-mouse IL-2 (clone JES6-5H4, BD Biosciences-US), or biotinylated rat anti-mouse IL-4 (BD Biosciences-US) at the concentration of 1 µg/mL, followed by streptavidin-HRP (3 µg/mL) for 2 h at room temperature. DAB substrate solution (100 µL, Invitrogen, Carlsbad, CA, USA) was added to the wells for developing color spots that were counted by ELISPOT automated reader.

### 2.8. Statistical Analysis

All results were presented as means ± SEM (standard error of mean). The statistical analysis was carried out by 1-way ANOVA and Tukey’s multiple comparison test using GraphPad Prism Software (GraphPad Software Inc., San Diego, CA, USA). *p* < 0.05 was considered as significant.

## 3. Results

### 3.1. Boost with Combination VLP+DNA Vaccines Prior to rMVA Enhances HIV Env gp120-Specific Antibody Responses 

An immunization regimen consisted of HIV DNA vaccine priming and rMVA boosting was previously shown to be effective in non-human primate studies and is now under clinical trials [12,13]. To further improve humoral and cellular immune responses in heterologous prime-boost regimen, we determined the effects of VLP and rMVA boosting immunizations. HIV-1 DNA vaccine encodes a chimeric hFlex-gp120 (HIV-1 89.6) as described [21]. VLP vaccine presents HIV-1 89.6 strain gp160 [25,26]. The rMVA expresses the hFlex-gp120 as determined by western blot (Figure 1). 

**Figure 2:**
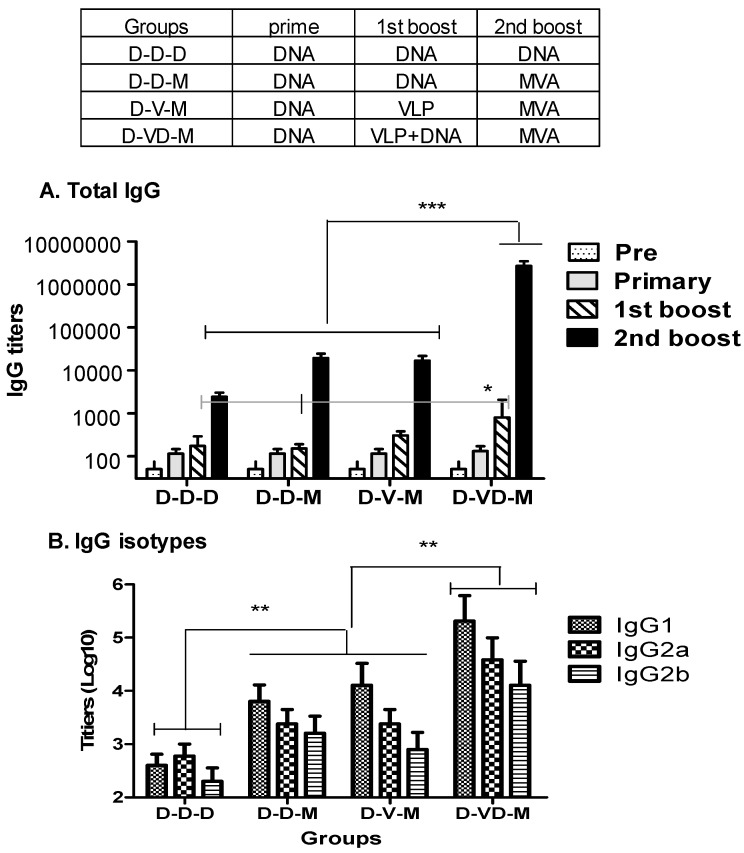
Vaccination groups and HIV-1 Env-specific IgG serum antibody titers. Top paned: Groups of mice (*n* = 10) received three sequential subcutaneous immunizations with vaccines by a four-week interval homo or heterologous prime-boost regimen. D-D-D: DNA (50 µg) prime—first boost—second boost with the same dose, D-D-M: DNA (50 µg) prime—DNA (50 µg) first boost—rMVA (1 × 10^7^ PFU) second boost, D-V-M: DNA (50 µg) prime—HIV VLP (50 µg) first boost—rMVA (1 × 10^7^ PFU) second boost, D-VD-M: DNA (50 µg) prime—combination VD first boost [HIV VLP (50 µg) + DNA (50 µg)]—rMVA (1 × 10^7^ PFU) second boost. (**A**) HIV-1 Env specific total IgG titers; (**B**) HIV-1 Env specific IgG isotype antibody titers at 12 days after the last vaccination. Antibody titer was independently measured for each mouse and statistically pooled to give the results depicted. For statistical analysis, One-way ANOVA and Tukey’s post-multiple comparison tests were performed. *: *p* < 0.5 between the D-VD-M and D-D-M (and D-D-D) groups after 1st boost. ***: *p* < 0.001, **: *p* < 0.01 between the comparing groups after second boost.

As shown in the Figure 2, all groups of mice were primed with a DNA vaccine. DNA priming induced a low level of antibodies specific to the HIV Env (Figure 2A). First boost was carried out with DNA (D) or VLP (V) vaccine or a mixture of VLP + DNA (VD) vaccines. First boosting with VLPs moderately increased the levels of antibodies primed by DNA vaccination by 2- to 3-fold (Figure 2A) whereas VLP boost immunization of VLP-primed mice effectively increased the levels of IgG antibodies by 10 folds. First boost of DNA vaccine was less effective in boosting antibody responses than boost immunization with a combination of VLP and DNA vaccines (Figure 2A). The group that received the first boost of a mixture of VD vaccines showed approximately four- to five-fold higher titers of HIV Env specific antibodies compared to the boosting with DNA alone groups, which is statistically significant (D-VD-M versus D-D-M and D-D-M, Figure 2A). After first boost, the D-VD-M group showed higher IgG antibody responses than those in the D-V-M group, but there was no significant difference between the these two groups (Figure 2A). 

To further enhance the immune responses to HIV Env, some groups received a second boost with rMVA expressing the hFlex-gp120 and a control group DNA vaccine as the second boost. The boost with rMVA significantly enhanced antibody responses by approximately 10 folds compared to the group boosted by DNA vaccine only (D-D-D vs. D-D-M, Figure 2A). There was no significant difference between the DNA and VLP boosted groups (D-D-M and D-V-M, Figure 2A). Most impressively, the group that was immunized with a mixture of VD vaccines (D-VD-M) boosted approximately 1000-fold increases in antibody titers, resulting in over 100-fold higher IgG titers than D-D-M and D-V-M groups (Figure 2A). These high IgG antibody titers were maintained for over 90 days after second boost.

To better understand the types of immune responses, antibody isotypes were determined. DNA vaccination (D-D-D) induced low levels of IgG1 and IgG2a isotypes with a trend of higher levels of IgG2a antibody but there was no significant difference between the two isotypes (Figure 2B). VLPs or rMVA boosting immunization induced significantly higher levels of IgG1, IgG2a, and IgG2b isotype antibodies compared to the D-D-D group (D-D-M, D-V-M; Figure 2B,C). The group of mice that received a mixture of VD vaccines induced the highest levels of IgG1, IgG2a, and IgG2b isotype antibodies (D-VD-M; Figure 2B). The neutralizing activity against pseudotyped HIV-1 SF162 strain in the D-VD-M group (540 ± 270) was approximately six-fold and 13-fold higher than those in the heterologous (90 ± 30 for D-D-M and D-V-M) and DNA only (40 ± 15 for D-D-D) groups, respectively, as measured by 50% reduction titers in plaque forming spots. 

Overall, these results suggest that first boost immunization with a combination of VLP and DNA (VD) vaccines prior to the second boost with rMVA significantly enhanced humoral IgG antibody immune responses to HIV Env protein by 100-fold compared to DNA or VLP only first boost groups (D-D-M, D-V-M). 

### 3.2. Boosting with Combination VLP + DNA Vaccines Prior to rMVA Enhances IFN-γ CD8 T Cells in Spleens 

**Figure 3:**
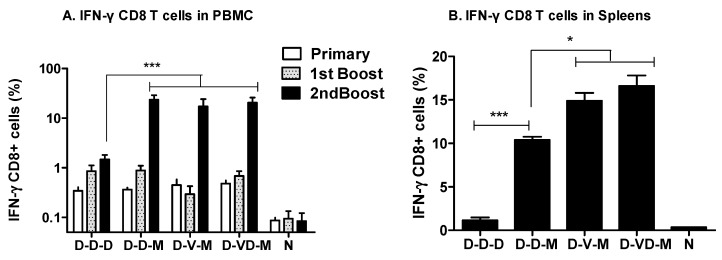
Boost with rMVA vaccine enhances IFN-γ positive CD8 T cell responses. (**A**) IFN-γ CD8 T cells in blood by an intracellular cytokine staining assay (ICC). PBMCs from the control and immunized mice were isolated at 12 days after each immunization. CD8^+^ T cells expressing intracellular IFN-γ in response to the gp120 V3 loop MHC-I peptide (ICC) was identified in a FACS Calibur. The average frequency of gp120-specific CD8^+^ T cells at primary and booster immunizations is shown in the histogram. The bars indicate values of standard variations (*n* = 5); (**B**) IFN-γ CD8 T cells in spleens by ICC. After 14 days second boost, spleen cells were stimulated using a pool of peptides. Groups are the same as described in the Figure 2 legend. ***: *p* < 0.001, *: *p* < 0.05 between the comparing groups after second boost.

It is important that HIV vaccination inducing antibody responses should also induce cellular immunity of the vaccines against intracellular pathogens such as HIV. As shown in Figure 3, we determined CD8 T cell responses in peripheral blood monocyte cells (PBMCs) by gating the intracellularly stained CD8 T cells after stimulating with an HIV-1 89.6 Env specific MHC I peptide [21]. Priming with DNA vaccines induced low but significant levels of CD8 T cells secreting IFN-γ (0.34 to 0.48%) compared to the naïve control (~0.09%). The first boost with DNA vaccines resulted in increases of IFN-γ secreting CD8 T cells by 2-fold whereas VLP boosting did not show any increase in IFN-γ secreting CD8 T cells (Figure 3A). Boost immunization with a mixture of VD vaccines showed an increased level in IFN-γ secreting CD8 T cells of PBMCs comparable to those in the DNA boost groups as determined by ICC. A second boost with DNA vaccine showed a moderate increase (~1.5%) in CD8 T cells secreting IFN-γ (D-D-D, Figure 3A). In contrast, rMVA boosting significantly increased the levels of CD8 T cells secreting IFN-γ up to a range of 17–23% (D-D-M, D-V-M, D-VD-M, Figure 3A).

To further extend the analysis of CD8 T cell responses, we analyzed the intracellularly stained CD8 T cells in splenocytes at two weeks after the rMVA boost immunization (Figure 3B). Immunization with DNA vaccines only (D-D-D) induced approximately 1.15% of CD8 T cells secreting IFN-γ out of total splenic CD8 T cells, which is significantly higher than that of the naïve control (0.15%). After rMVA boost, the levels of IFN-γ secreting CD8 T cells were significantly increased up to 10% (D-D-M, Figure 3B). Interestingly, the groups of mice that were first boosted with VLPs or a mixture of VD showed significantly higher levels of IFN-γ CD8 T cells up to 14–17% (D-V-M, D-VD-M, Figure 3B) compared to DNA vaccines only immunizations (D-D-D). The group of VD combination boost showed a trend to induce higher levels of IFN-γ+ splenic CD8 T cell responses than those in the VLP boost group (D-V-M) although there is no statistical difference between the two groups after second boost with rMVA. Taken together, these results suggest that first boost immunization with a combination VD vaccine (D-VD-M) significantly enhances CD8 T cells secreting IFN-γ cytokine in lymphoid organs such as spleen compared to the DNA boost (D-D-M) prior to MVA second boost immunization.

### 3.3. Boost with a Combination of VLP and DNA Vaccine Prior to rMVA Boost Effectively Induces IFN-γ CD4^+^ T Cells 

**Figure 4:**
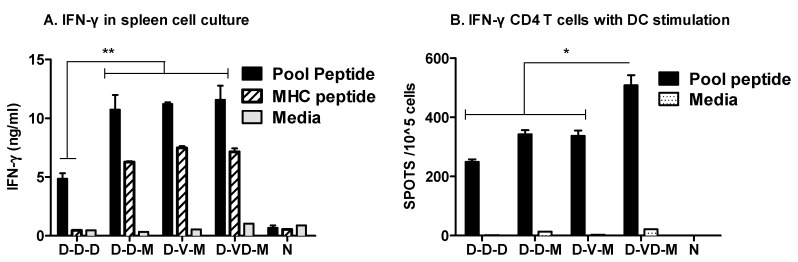
IFN-γ production from spleen cells or IFN-γ+ spots of CD4^+^ T cells in vitro. (**A**) IFN-γ production from spleen cell cultures. IFN-γ cytokine levels in spleen cell (1 × 10^6^) culture supernatants were determined by using cytokine ELISA after stimulation with a pool of peptides or MHC I restricted peptide; (**B**) IFN-γ secreting spots in CD4 T cells + DC cultures. CD4^+^ (1 × 10^5^) T cells (purified from spleen cells) in the presence of DCs (1 × 10^4^) loaded with a pool of peptides were plated in 96 well ELISPOT plates coated with anti IFN-γ antibodies in triplicates. Spleen cells were collected at 14 days after second boost. Groups are the same as described in the Figure 2 legend. **: *p* < 0.01, *: *p* < 0.05 between the comparing groups in the pooled peptides after second boost.

As an additional measure of IFN-γ producing T cell responses, we determined the levels of IFN-γ cytokine secreted into culture supernatants. Spleens were collected from immunized or naïve mice and single cell suspensions were stimulated with an HIV-1 89.6 Env specific MHC I peptide or a pool of peptides covering the entire HIV-1 89.6 Env protein (Figure 4A). Significantly higher levels of IFN-γ in response to an MHC-I peptide were found to be secreted into the culture media of splenocytes from rMVA boost groups (D-D-M, D-V-M, D-VD-M) but not DNA only immunized mice. When a pool of peptides was used as a stimulator, enhanced levels of IFN-γ were found in culture media of rMVA boost immunized groups whereas the DNA only vaccination group induced moderate levels of IFN-γ (Figure 4A). 

CD4^+^ T cells are also an important immune component for helping generation and maintenance of effective cytotoxic CD8 T cells and antibody-producing B cells. Purified CD4^+^ T cells were stimulated with DCs loaded with a pool of peptides covering the whole HIV-1 89.6 Env protein (Figure 4B). DCs loaded with a pool of peptides effectively stimulated CD4 T cells collected from immunized mice but not from naïve mice. The group boosted with a mixture of VD vaccines prior to rMVA second boost induced higher levels of IFN-γ producing CD4 T cell spots (~500/1 × 10^5^ CD4 T cells) in response to the stimulation with a pool of peptides than those in the DNA boost (D-D-D, D-D-M) and VLP boost (D-V-M) groups. Therefore, these results suggest that boost immunization with a combination of DNA and VLP vaccines prior to rMVA is effective for inducing IFN-γ CD4 T cell responses to a broader range of peptide epitopes in BALB/c mice. 

### 3.4. Combination of VLP and DNA Vaccination Effectively Induces Splenocyte Proliferation Responses 

**Figure 5:**
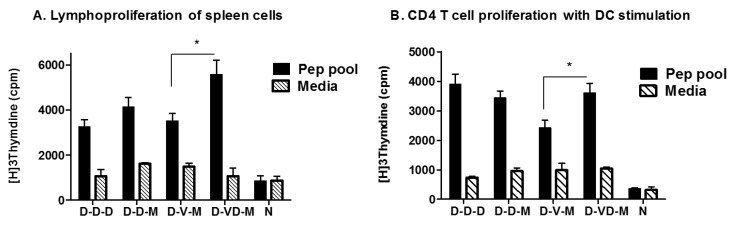
Proliferative responses of whole spleen cells or CD4^+^ T cells. Spleens isolated from each group of mice at 14 days after a second boost were used for single cell preparation and to isolate CD4^+^ cells. The cells were stimulated by a pool of peptides for 3 days of incubation, loaded with 3[H]-thymidine overnight and incorporated radioactivity was measured after lysis. Env-specific spleen cell and CD4^+^ T cell proliferation was observed. Spleen cells (1 × 10^6^) and CD4^+^ (1 × 10^5^) cells in the presence of DCs (1 × 10^4^) at ratio of 1:10 were plated in 96 well plate in triplicates. *: *p* < 0.05 between the comparing groups (D-V-M and D-VD-M) in the peptide pool after second boost.

Lymphocyte proliferative assay is often used as an indicator for CD4 T cell responses. Stimulation of whole splenocytes with a pool of HIV Env peptides produced significant responses of cells incorporating H3-labeled thymidine compared to the naïve or medium only controls, indicating HIV Env antigen specific T cell proliferation (Figure 5). In contrast to IFN-γ secreting CD8 T cell responses, the combined VLP + DNA (D-VD-M) vaccination groups showed meaningful proliferation index of splenocytes by over three folds, which are higher than other groups (Figure 5A). To further define the CD4^+^ T cell responses, purified CD4 T cells were stimulated with dendritic cells (DCs) loaded with a pool of HIV Env peptides. DNA boost (D-D-D, D-D-M) and combination VLP + DNA boost (D-VD-M) groups displayed positive proliferation index whereas the VLP boost group (D-V-M) did not show stimulation index of 3 in proliferative responses of splenic CD4^+^ T cells from (Figure 5B). 

### 3.5. Boost with Combination VLP + DNA Vaccines Prior to rMVA Is Effective in Induceing IL-4 CD4^+^ T Cells 

**Figure 6:**
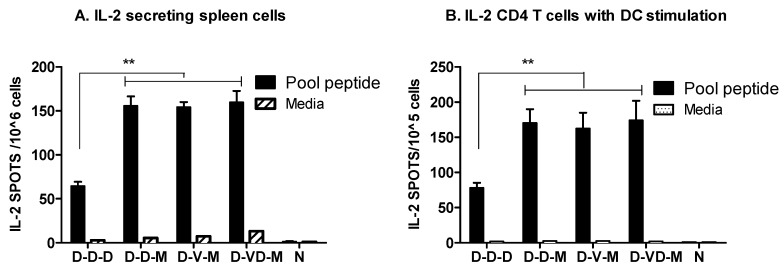
IL-2 secreting cell spots from spleen cells or from CD4 T cells plus DCs in vitro. (**A**) IL-2 secreting cell spots from spleen cell cultures. The levels of IL-2 secreting cell spots in spleen cell (1 × 10^6^) cultures were determined by IL-2 cytokine ELISPOT after stimulation with a pool of peptides; (**B**) IL-2 secreting spots in CD4 T cells plus DC cultures. CD4^+^ T (1 × 10^5^) cells (purified from spleen cells) in the presence of DCs (1 × 10^4^) loaded with a pool of peptides were plated in 96 well ELISPOT plates coated with anti-IL-2 antibodies in triplicates. Spleen cells were collected at 14 days after second boost. Groups are the same as described in the Figure 2 legend. **: *p* < 0.001 between the comparing groups in the peptide pool after second boost.

IL-2 is known to be an important cytokine produced by activated T cells for controlling viral infection. Whole spleen cells or purified CD4^+^ T cells were stimulated with a pool of HIV Env peptides. DNA or VLP immunization followed by rMVA boost (D-D-M, D-V-M, D-VD-M) induced substantial numbers of spots secreting IL-2 cytokine, which is approximately 2.5-fold higher than the DNA vaccine only group (Figure 6A). The IL-2 secreting spots were similar between the whole splenocytes and purified CD4 T cells (Figure 6B). 

**Figure 7:**
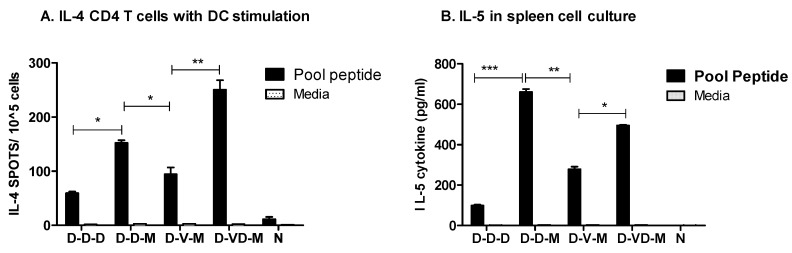
IL-4 or IL-5 cytokine producing cellular responses. (**A**) IL-4 secreting cell spots in CD4 T cell stimulation. CD4^+^ T (1 × 10^5^) cells (from spleen cells) in the presence of DCs (1 × 10^4^) loaded with a pool of peptides were plated in 96-well ELISPOT plates coated with anti-IL-4 antibodies in triplicates; (**B**) IL-5 production from spleen cell cultures. IL-5 cytokine levels in spleen cell (1 × 10^6^) culture supernatants were determined by using IL-5 cytokine ELISA after stimulation with a pool of peptides. Groups are the same as described in the Figure 2 legend. *: *p* < 0.05, **: *p* < 0.001, ***: *p* < 0.0001 between the comparing groups in the peptide pool after second boost.

We also found that boost with rMVA in combination with DNA or combination VD vaccines induced higher levels of IL-4 spots than DNA only vaccination in the purified CD4 T cells in the co-culture of preloaded DCs with a pool of peptides (Figure 7A). In particular, the group of mice which received a mixture of VD vaccine boost prior to rMVA produced higher levels of IL-4 cytokine secreting CD4 T cell responses under DC stimulation compared to those in DNA or VLP boost groups (D-D-D, D-D-M, D-V-M, Figure 7A). We observed that significant amounts of IL-5 cytokine were secreted into culture media of spleen cell cultures, resulting in the highest level in the DNA boost group prior to rMVA second boost in response to the stimulation with a pool of HIV-1 Env peptides (Figure 7B). 

Taken together, boost with a combination of VLP and DNA vaccines prior to rMVA second boost was found to be effective in inducing IL-4 secreting splenic CD4^+^ T cell responses under in vitro DC stimulation. Also, DNA or combination VD vaccine followed by rMVA boost vaccination were effective in inducing T cells capable of secreting various cytokines in responses to antigenic stimulation.

## 4. Discussion

One of the promising vaccine candidates against HIV-1 or simian immunodeficiency virus is a regimen of DNA-rMVA prime-boost vaccination as demonstrated in preclinical studies [12,18,24,29,30,31,32]. Also, this strategy of DNA-rMVA vaccination against HIV was shown to be safe and immunogenic in clinical trials [14,33]. This study investigated the effects of first boosting with VLPs or a mixture of VLP and DNA (VD) vaccines on humoral and cellular immune responses in comparison with DNA only or DNA prime rMVA boost (D-D-M) vaccinations. The magnitude of boosting antibody responses by either DNA or VLP vaccine alone was within a few folds or no significant increases. In contrast, boost immunization with a mixture of DNA plus VLP vaccines was found to significantly enhance the primed antibody responses. Consistent with these results, increased antibody and CD8 T cell responses were observed with prime-boost with combination DNA and VLP vaccines in a previous study with experimental settings [34]. Buonaguro et al reported a comparative study demonstrating that heterologous prime-boost immunization with DNA and VLP vaccines induced higher levels of HIV Env specific antibodies compared to the homologous prime-boost immunization with either vaccine alone [35]. In a previous study, 2 times immunizations of mice with recombinant Semliki Forest virus expressing HIV-1 Env protein and then second boosted by HIV-1 Env glycoproteins (10 µg) induced an average 10^4^ titers of Env specific IgG antibodies [36]. A phase 1 safety and immunogenicity study of rMVA expressing HIV-1 env, gag, pol, nef, and tat genes reported an average of binding IgG titers of 10^3^ after 3 immunizations of healthy adults with 2.5 × 10^8^ infectious particles [37]. DNA priming and boost with recombinant fowl pox viral vectored vaccines expressing HIV Env clade A/E were not very effective in inducing Env specific antibody responses in a clinical trial [38]. In contrast, influenza VLPs containing hemagglutinin were found to induce antibody responses conferring protective immunity even after a single dose vaccination [39,40]. Taken together, our present and other previous studies suggest that HIV Env might be intrinsically weakly immunogenic compared to other viral glycoproteins such as influenza virus. 

Homologous prime boost vaccination with HIV VLP (V-V) induced higher levels of HIV Env specific IgG antibodies than those with D-D or D-V vaccination. It is not clear why DNA-primed mice responded only moderately to VLP boost. However, significant differential effects were observed after the second boost. The levels of HIV Env antibodies were boosted by approximately 10-fold after the third DNA vaccination. The second boost by rMVA resulted in approximately 100-fold increases in HIV Env specific antibody levels as observed in mice with a D-D-M or D-V-M heterologous prime-boost vaccination strategy. Also, a similar pattern of boosting effects by rMVA was reported in non-human primate models at a differential level [32]. The striking result in this study was that the first boost of DNA-primed mice with a mixture of DNA plus VLP vaccines induced over a 1000-fold increase in HIV Env specific antibody responses after the second boost with rMVA. The resulting antibody levels were approximately 100-fold higher than those induced by D-D-M or D-V-M prime boost vaccination. This 2 log magnitudes of parallel boost effects was observed when DNA-rMVA prime-boosted monkeys were challenged with a pathogenic SHIV live virus [32]. 

The mechanisms are not clear how a combination form of DNA and VLP vaccines but not DNA or VLP vaccine alone resulted in highly effective boosting effects after rMVA vaccination. It is speculated that mixing DNA and VLPs might represent a highly immunogenic form resulting in more effective priming of the immune system. A previous study demonstrated that DNA plasmids were bound to VLPs when mixed together [34]. VLPs were shown to effectively stimulate dendritic cells [41,42,43,44]. Similar activation of dendritic cells was observed by a mixture DNA and VLP vaccines but not by DNA vaccine alone [34]. Also, it was demonstrated that dendritic cells can cross-present VLPs via an endosome-to-cytosol processing pathway [45]. Liposomes or VLPs encapsulating DNA vaccines were shown to induce effective immune responses probably due to the enhanced uptake of DNA vaccines by host cells [46,47]. In a recent study, boost vaccination with VLPs expressing simian immunodeficiency virus gp160 Env enhanced the breadth of DNA-rMVA-induced antibody responses in rhesus macaques [24]. In this study, we found that combination VD boost vaccination appears to broaden the CD8 T cell responses to a pool of peptides. Further studies are needed to better understand the working mechanisms of a mixture of DNA plus VLP vaccines. 

It is highly desirable but rare for a vaccine to induce effectively both humoral and cellular immune responses. DNA vaccines are relatively effective in inducing T cell immunity, whereas protein subunit–based vaccines such as VLPs are effective in inducing humoral responses [34,48]. It was also demonstrated that the MVA-only vaccine induced 10-fold lower vaccine-specific T cell responses but 10-fold-higher titers of HIV Env specific binding antibody compared to the DNA-rMVA vaccine [32]. To enhance protective humoral immunity induced by DNA-rMVA vaccination, previous studies demonstrated the effects of granulocyte-macrophage colony-stimulating factor (GM-CSF) on inducing antibody responses and an inverse correlation between the levels of non-neutralizing antibodies with high avidity and peaks of viremia [18,19,20]. In the present study, enhanced IgG antibody responses were induced by heterologous prime-boost vaccination (D-VD-M) together with increasing T cell response or at least without reduction in inducing T cell immunity. Compared to DNA-rMVA vaccination that is a regimen used extensively in non-human primate studies, boost with a VD combination followed by rMVA vaccination induced comparable or higher levels T cell responses including IFN-γ expressing PBMC, IFN-γ expressing splenic CD8 T and CD4 T cells, and lymphocyte proliferative responses, as well as IL-2 and IL-4 cytokine expressing CD4 T cells. Interestingly, the highest levels of CD4 T cells secreting IFN-γ or IL-4 were observed in mice that received a D-VD-M vaccination in response to a pool of peptides, indicating potentially a greater breadth of CD4 T cell responses. IL-5 secretion was highest in the D-D-M group followed by the D-VD-M and D-V-M vaccination groups, and lowest in the D-D-D group. 

We observed differential effects of rMVA boosting on inducing cellular immune responses. Approximately 10-fold or higher levels of IFN-γ producing CD8 T cells in responses to an immunodominant MHC I peptide stimulation were induced in the groups with rMVA boost when compared to the DNA vaccine only (D-D-D) group. This fold increase in IFN-γ positive CD8 T cells was similarly observed in previous studies [32,49]. Compared to the responding CD8 T cells, IFN-γ or IL-2 producing CD4 T cells in response to a pool of peptide stimulation were substantially induced by boost vaccination with DNA vaccines, which was only two- or three-fold less than those by rMVA boost. Consistent with our results, a previous study has shown that rMVA boost preferentially increased the number of IFN- γ producing CD8 T cells compared to the CD4 T cells [49]. Nonetheless, DNA priming is known to be important since rMVA only vaccine was less effective in stimulating T cell responses than DNA-rMVA vaccination [32]. In addition, DNA vaccine prime and boost immunization induced higher levels of CD8 T cells but lower IgG humoral responses than DNA-VLPs or VLPs prime boost immunizations. In particular, the present study revealed that DNA boost was as effective as rMVA boost in CD4^+^ T cell proliferative responses. 

## 5. Conclusions

In summary, the results from this study provide evidence that boost vaccination with a mixture of DNA and VLPs prior to rMVA second boost in combination with DNA prime provide an effective approach for inducing enhanced levels of HIV Env specific antibody responses as well as CD8 and CD4 T cell responses.

## References

[1] Desrosiers R.C. (2004). Prospects for an AIDS vaccine. Nat. Med..

[2] Baba T.W., Liska V., Khimani A.H., Ray N.B., Dailey P.J., Penninck D., Bronson R., Greene M.F., McClure H.M., Martin L.N. (1999). Live attenuated, multiply deleted simian immunodeficiency virus causes AIDS in infant and adult macaques. Nat. Med..

[3] Sekaly R.P. (2008). The failed HIV Merck vaccine study: A step back or a launching point for future vaccine development?. J. Exp. Med..

[4] Valentine L.E., Watkins D.I. (2008). Relevance of studying T cell responses in SIV-infected rhesus macaques. Trends Microbiol..

[5] Watkins D.I., Burton D.R., Kallas E.G., Moore J.P., Koff W.C. (2008). Nonhuman primate models and the failure of the Merck HIV-1 vaccine in humans. Nat. Med..

[6] Rerks-Ngarm S., Pitisuttithum P., Nitayaphan S., Kaewkungwal J., Chiu J., Paris R., Premsri N., Namwat C., de Souza M., Adams E. (2009). Vaccination with ALVAC and AIDSVAX to prevent HIV-1 infection in Thailand. N. Engl. J. Med..

[7] Blumberg R.S., Paradis T., Hartshorn K.L., Vogt M., Ho D.D., Hirsch M.S., Leban J., Sato V.L., Schooley R.T. (1987). Antibody-dependent cell-mediated cytotoxicity against cells infected with the human immunodeficiency virus. J. Infect. Dis..

[8] Evans L.A., Thomson-Honnebier G., Steimer K., Paoletti E., Perkus M.E., Hollander H., Levy J.A. (1989). Antibody-dependent cellular cytotoxicity is directed against both the gp120 and gp41 envelope proteins of HIV. AIDS.

[9] Blue C.E., Spiller O.B., Blackbourn D.J. (2004). The relevance of complement to virus biology. Virology.

[10] Jayasekera J.P., Moseman E.A., Carroll M.C. (2007). Natural antibody and complement mediate neutralization of influenza virus in the absence of prior immunity. J. Virol..

[11] Xu Y., Zhang C., Jia L., Wen C., Liu H., Wang Y., Sun Y., Huang L., Zhou Y., Song H. (2009). A novel approach to inhibit HIV-1 infection and enhance lysis of HIV by a targeted activator of complement. Virol. J..

[12] Iyer S.S., Amara R.R. (2014). DNA/MVA Vaccines for HIV/AIDS. Vaccines (Basel).

[13] Hanke T., McMichael A.J., Mwau M., Wee E.G., Ceberej I., Patel S., Sutton J., Tomlinson M., Samuel R.V. (2002). Development of a DNA-MVA/HIVA vaccine for Kenya. Vaccine.

[14] Goepfert P.A., Elizaga M.L., Sato A., Qin L., Cardinali M., Hay C.M., Hural J., DeRosa S.C., DeFawe O.D., Tomaras G.D. (2011). Phase 1 safety and immunogenicity testing of DNA and recombinant modified vaccinia Ankara vaccines expressing HIV-1 virus-like particles. J. Infect. Dis..

[15] Goulder P.J., Rowland-Jones S.L., McMichael A.J., Walker B.D. (1999). Anti-HIV cellular immunity: Recent advances towards vaccine design. AIDS.

[16] Schmitz J.E., Kuroda M.J., Santra S., Sasseville V.G., Simon M.A., Lifton M.A., Racz P., Tenner-Racz K., Dalesandro M., Scallon B.J. (1999). Control of viremia in simian immunodeficiency virus infection by CD8+ lymphocytes. Science.

[17] Levy J.A., Mackewicz C.E., Barker E. (1996). Controlling HIV pathogenesis: The role of the noncytotoxic anti-HIV response of CD8+ T cells. Immunol. Today.

[18] Lai L., Vodros D., Kozlowski P.A., Montefiori D.C., Wilson R.L., Akerstrom V.L., Chennareddi L., Yu T., Kannanganat S., Ofielu L. (2007). GM-CSF DNA: An adjuvant for higher avidity IgG, rectal IgA, and increased protection against the acute phase of a SHIV-89.6P challenge by a DNA/MVA immunodeficiency virus vaccine. Virology.

[19] Robinson H.L., Montefiori D.C., Villinger F., Robinson J.E., Sharma S., Wyatt L.S., Earl P.L., McClure H.M., Moss B., Amara R.R. (2006). Studies on GM-CSF DNA as an adjuvant for neutralizing Ab elicited by a DNA/MVA immunodeficiency virus vaccine. Virology.

[20] Zhao J., Lai L., Amara R.R., Montefiori D.C., Villinger F., Chennareddi L., Wyatt L.S., Moss B., Robinson H.L. (2009). Preclinical studies of human immunodeficiency virus/AIDS vaccines: Inverse correlation between avidity of anti-Env antibodies and peak postchallenge viremia. J. Virol..

[21] Sailaja G., Husain S., Nayak B.P., Jabbar A.M. (2003). Long-term maintenance of gp120-specific immune responses by genetic vaccination with the HIV-1 envelope genes linked to the gene encoding Flt-3 ligand. J. Immunol..

[22] Kwa S., Sadagopal S., Shen X., Hong J.J., Gangadhara S., Basu R., Victor B., Iyer S.S., LaBranche C.C., Montefiori D.C. (2015). CD40L-adjuvanted DNA/modified vaccinia virus Ankara simian immunodeficiency virus (SIV) vaccine enhances protection against neutralization-resistant mucosal SIV infection. J. Virol..

[23] Kannanganat S., Wyatt L.S., Gangadhara S., Chamcha V., Chea L.S., Kozlowski P.A., LaBranche C.C., Chennareddi L., Lawson B., Reddy P.B. (2016). High Doses of GM-CSF Inhibit Antibody Responses in Rectal Secretions and Diminish Modified Vaccinia Ankara/Simian Immunodeficiency Virus Vaccine Protection in TRIM5alpha-Restrictive Macaques. J. Immunol..

[24] Iyer S.S., Gangadhara S., Victor B., Shen X., Chen X., Nabi R., Kasturi S.P., Sabula M.J., Labranche C.C., Reddy P.B. (2016). Virus-Like Particles Displaying Trimeric Simian Immunodeficiency Virus (SIV) Envelope gp160 Enhance the Breadth of DNA/Modified Vaccinia Virus Ankara SIV Vaccine-Induced Antibody Responses in Rhesus Macaques. J. Virol..

[25] Quan F.S., Sailaja G., Skountzou I., Huang C., Vzorov A., Compans R.W., Kang S.M. (2007). Immunogenicity of virus-like particles containing modified human immunodeficiency virus envelope proteins. Vaccine.

[26] Sailaja G., Skountzou I., Quan F.S., Compans R.W., Kang S.M. (2007). Human immunodeficiency virus-like particles activate multiple types of immune cells. Virology.

[27] Belyakov I.M., Wyatt L.S., Ahlers J.D., Earl P., Pendleton C.D., Kelsall B.L., Strober W., Moss B., Berzofsky J.A. (1998). Induction of a mucosal cytotoxic T-lymphocyte response by intrarectal immunization with a replication-deficient recombinant vaccinia virus expressing human immunodeficiency virus 89.6 envelope protein. J. Virol..

[28] Takahashi H., Cohen J., Hosmalin A., Cease K.B., Houghten R., Cornette J.L., DeLisi C., Moss B., Germain R.N., Berzofsky J.A. (1988). An immunodominant epitope of the human immunodeficiency virus envelope glycoprotein gp160 recognized by class I major histocompatibility complex molecule-restricted murine cytotoxic T lymphocytes. Proc. Natl. Acad. Sci. USA.

[29] Chamcha V., Kannanganat S., Gangadhara S., Nabi R., Kozlowski P.A., Montefiori D.C., LaBranche C.C., Wrammert J., Keele B.F., Balachandran H. (2016). Strong, but Age-Dependent, Protection Elicited by a Deoxyribonucleic Acid/Modified Vaccinia Ankara Simian Immunodeficiency Virus Vaccine. Open Forum Infect. Dis..

[30] Nigam P., Earl P.L., Americo J.L., Sharma S., Wyatt L.S., Edghill-Spano Y., Chennareddi L.S., Silvera P., Moss B., Robinson H.L. (2007). DNA/MVA HIV-1/AIDS vaccine elicits long-lived vaccinia virus-specific immunity and confers protection against a lethal monkeypox challenge. Virology.

[31] Smith J.M., Amara R.R., Campbell D., Xu Y., Patel M., Sharma S., Butera S.T., Ellenberger D.L., Yi H., Chennareddi L. (2004). DNA/MVA vaccine for HIV type 1: Effects of codon-optimization and the expression of aggregates or virus-like particles on the immunogenicity of the DNA prime. AIDS Res. Hum. Retrovir..

[32] Amara R.R., Villinger F., Staprans S.I., Altman J.D., Montefiori D.C., Kozyr N.L., Xu Y., Wyatt L.S., Earl P.L., Herndon J.G. (2002). Different patterns of immune responses but similar control of a simian-human immunodeficiency virus 89.6P mucosal challenge by modified vaccinia virus Ankara (MVA) and DNA/MVA vaccines. J. Virol..

[33] Goepfert P.A., Elizaga M.L., Seaton K., Tomaras G.D., Montefiori D.C., Sato A., Hural J., DeRosa S.C., Kalams S.A., McElrath M.J. (2014). Specificity and 6-month durability of immune responses induced by DNA and recombinant modified vaccinia Ankara vaccines expressing HIV-1 virus-like particles. J. Infect. Dis..

[34] Ye L., Wen Z., Dong K., Pan L., Bu Z., Compans R.W., Zhang H., Yang C. (2010). Immunization with a Mixture of HIV Env DNA and VLP Vaccines Augments Induction of CD8 T Cell Responses. J. Biomed. Biotechnol..

[35] Buonaguro L., Devito C., Tornesello M.L., Schroder U., Wahren B., Hinkula J., Buonaguro F.M. (2007). DNA-VLP prime-boost intra-nasal immunization induces cellular and humoral anti-HIV-1 systemic and mucosal immunity with cross-clade neutralizing activity. Vaccine.

[36] Forsell M.N., Li Y., Sundback M., Svehla K., Liljestrom P., Mascola J.R., Wyatt R., Karlsson Hedestam G.B. (2005). Biochemical and immunogenic characterization of soluble human immunodeficiency virus type 1 envelope glycoprotein trimers expressed by semliki forest virus. J. Virol..

[37] Vasan S., Schlesinger S.J., Chen Z., Hurley A., Lombardo A., Than S., Adesanya P., Bunce C., Boaz M., Boyle R. (2010). Phase 1 safety and immunogenicity evaluation of ADMVA, a multigenic, modified vaccinia Ankara-HIV-1 B’/C candidate vaccine. PLoS ONE.

[38] Hemachandra A., Puls R.L., Sirivichayakul S., Kerr S., Thantiworasit P., Ubolyam S., Cooper D.A., Emery S., Phanuphak P., Kelleher A. (2010). An HIV-1 clade A/E DNA prime, recombinant fowlpox virus boost vaccine is safe, but non-immunogenic in a randomized phase 1/11a trial in Thai volunteers at low risk of HIV infection. Hum. Vaccines.

[39] Quan F.S., Kim Y.C., Yoo D.G., Compans R.W., Prausnitz M.R., Kang S.M. (2009). Stabilization of influenza vaccine enhances protection by microneedle delivery in the mouse skin. PLoS ONE.

[40] Song J.M., Hossain J., Yoo D.G., Lipatov A.S., Davis C.T., Quan F.S., Chen L.M., Hogan R.J., Donis R.O., Compans R.W. (2010). Protective immunity against H5N1 influenza virus by a single dose vaccination with virus-like particles. Virology.

[41] Bosio C.M., Moore B.D., Warfield K.L., Ruthel G., Mohamadzadeh M., Aman M.J., Bavari S. (2004). Ebola and Marburg virus-like particles activate human myeloid dendritic cells. Virology.

[42] Buonaguro L., Tornesello M.L., Tagliamonte M., Gallo R.C., Wang L.X., Kamin-Lewis R., Abdelwahab S., Lewis G.K., Buonaguro F.M. (2006). Baculovirus-derived human immunodeficiency virus type 1 virus-like particles activate dendritic cells and induce ex vivo T-cell responses. J. Virol..

[43] Lenz P., Day P.M., Pang Y.Y., Frye S.A., Jensen P.N., Lowy D.R., Schiller J.T. (2001). Papillomavirus-like particles induce acute activation of dendritic cells. J. Immunol..

[44] Zhang R., Li M., Chen C., Yao Q. (2004). SHIV virus-like particles bind and activate human dendritic cells. Vaccine.

[45] Moron V.G., Rueda P., Sedlik C., Leclerc C. (2003). In vivo, dendritic cells can cross-present virus-like particles using an endosome-to-cytosol pathway. J. Immunol..

[46] Takamura S., Niikura M., Li T.C., Takeda N., Kusagawa S., Takebe Y., Miyamura T., Yasutomi Y. (2004). DNA vaccine-encapsulated virus-like particles derived from an orally transmissible virus stimulate mucosal and systemic immune responses by oral administration. Gene Ther..

[47] Gurunathan S., Klinman D.M., Seder R.A. (2000). DNA vaccines: Immunology, application, and optimization. Annu. Rev. Immunol..

[48] Quan F.S., Yoo D.G., Song J.M., Clements J.D., Compans R.W., Kang S.M. (2009). Kinetics of immune responses to influenza virus-like particles and dose-dependence of protection with a single vaccination. J. Virol..

[49] Liu J., Hellerstein M., McDonnel M., Amara R.R., Wyatt L.S., Moss B., Robinson H.L. (2007). Dose-response studies for the elicitation of CD8 T cells by a DNA vaccine, used alone or as the prime for a modified vaccinia Ankara boost. Vaccine.

